# Detection of the Invasive Mosquito Species *Aedes* (*Stegomyia*) *albopictus* (Diptera: Culicidae) in Portugal

**DOI:** 10.3390/ijerph15040820

**Published:** 2018-04-21

**Authors:** Hugo Costa Osório, Líbia Zé-Zé, Maria Neto, Sílvia Silva, Fátima Marques, Ana Sofia Silva, Maria João Alves

**Affiliations:** 1 National Institute of Health Doutor Ricardo Jorge, Centre for Vectors and Infectious Diseases Research, Avenida da Liberdade 5, 2965-575 Águas de Moura, Portugal; libia.zeze@insa.min-saude.pt (L.Z.-Z.); m.joao.alves@insa.min-saude.pt (M.J.A.); 2 Instituto de Saúde Ambiental, Faculdade de Medicina da Universidade de Lisboa, Av. Prof. Egas Moniz, Ed. Egas Moniz, Piso 0, Ala C, 1649-028 Lisboa, Portugal; 3 Biosystems and Integrative Sciences Institute (BioISI), Edificio TecLabs, Campus da FCUL, Campo Grande, 1749-016 Lisboa, Portugal; 4 Administração Regional de Saúde do Norte, I.P., Departamento de Saúde Pública, Rua Anselmo Braamcamp, 144, 4000-078 Porto, Portugal; mneto@arsnorte.min-saude.pt (M.N.); scsilva@arsnorte.min-saude.pt (S.S.); 5 Agrupamento de Centros de Saúde de Vale de Sousa Sul—Unidade de Saúde Pública, Avenida Comendador Abílio Seabra, 104, 4580-029 Paredes, Portugal; asparedes@csparedes.min-saude.pt (F.M.); asgrmsilva@arsnorte.min-saude.pt (A.S.S.)

**Keywords:** *Aedes albopictus*, invasive mosquito species, DNA barcoding, REVIVE, arboviruses, Portugal

## Abstract

The Asian tiger mosquito *Aedes albopictus* is an invasive mosquito originating from the Asia-Pacific region. This species is of major concern to public and veterinary health because of its vector role in the transmission of several pathogens, such as chikungunya, dengue, and Zika viruses. In Portugal, a National Vector Surveillance Network (REde de VIgilância de VEctores—REVIVE) is responsible for the surveillance of autochthonous, but also invasive, mosquito species at points of entry, such as airports, ports, storage areas, and specific border regions with Spain. At these locations, networks of mosquito traps are set and maintained under surveillance throughout the year. In September 2017, *Ae. albopictus* was detected for the first time in a tyre company located in the North of Portugal. Molecular typing was performed, and a preliminary phylogenetic analysis indicated a high similarity with sequences of *Ae. albopictus* collected in Europe. A prompt surveillance response was locally implemented to determine its dispersal and abundance, and adult mosquitoes were screened for the presence of arboviral RNA. A total of 103 specimens, 52 immatures and 51 adults, were collected. No pathogenic viruses were detected. Despite the obtained results suggest low abundance of the population locally introduced, the risk of dispersal and potential establishment of *Ae. albopictus* in Portugal has raised concern for autochthonous mosquito-borne disease outbreaks.

## 1. Introduction

*Aedes albopictus* (Skuse, 1894) is an invasive mosquito species of major concern to public health because of its vector role in the transmission of several arboviruses, such as chikungunya, dengue, and Zika [1]. This species has spread globally during the last four decades from its original distribution range in the Asia-Pacific region. This worldwide expansion was facilitated mainly by human trade via passive transport of eggs on used tyres and ornamental plants (such as “lucky bamboo”), together with passive transportation of adult mosquitoes by public and private ground transport from heavily infested areas [2].

In Europe, this species was first detected in Albania in 1979 [3]. Since its finding in Italy in 1990, *Ae. albopictus* is known to be spreading in several European countries such as the Balkan countries, Greece, France, and Spain, with demonstration of its presence in the latter country almost everywhere along the Mediterranean coast [4,5,6,7,8]. At present, *Ae. albopictus* has already been recorded in 25 European countries and has become established in 19 of them [9,10]. Outbreaks of mosquito-borne diseases related to *Ae. albopictus*, which were historically confined to tropical environments, have been recorded in Europe since 2007, when the first outbreak of chikungunya virus occurred in Italy [11]. This was followed by an outbreak of dengue virus in France in 2014 and by the recent outbreaks of chikungunya in France and Italy in 2017 [12,13].

Portugal has always been on the list of the most likely regions for the introduction and establishment of *Ae. albopictus* [2], and the species' characteristics have already been included in the taxonomic identification keys of Portuguese mosquitoes [14].

In Portugal, a National Vector Surveillance Network—REVIVE (REde de VIgilância de VEctores)—has been in operation since 2008 under the custody of the Portuguese Ministry of Health [15]. The actual REVIVE plan includes the following institutions: General Directorate of Health (DGS), the five Regional Health Administrations (ARS), namely Algarve, Alentejo, Lisboa e Vale do Tejo, Centro and Norte, the National Institute of Health Doutor Ricardo Jorge (INSA), and, in the outermost regions, the Institute of Health Administration of Madeira and the Regional Health Directorate of Azores. REVIVE is responsible for the nationwide surveillance of the most significant hematophagous arthropods in public health: mosquitoes, ticks, and sandflies. Surveillance of invasive mosquito species, such as *Aedes aegypti* and *Ae. albopictus*, and screening of field-collected mosquitoes for arboviruses is regularly performed. At airports, ports, storage areas, and specific border regions with Spain, monitoring takes place throughout the year with the commitment of local and regional authorities. *Aedes aegypti*, first recorded in the Autonomous Portuguese region of Madeira in 2005 [16], where it is now established, is currently surveyed within REVIVE [15]. Since 2008, the REVIVE operational programme did not show any evidence of the presence of *Ae. aegypti* outside Madeira island, and *Ae. albopictus* was never identified in the mainland or islands. Notwithstanding, considering that nowadays *Ae. albopictus* is the most invasive mosquito species and that its expansion in Europe is still ongoing, new introductions are very likely to occur in new geographic areas by global trade, making national surveillance essential at the points of entry [1]. Only in this way, a rapid implementation of control measures would be possible, as an effort to eliminate this vector species and to prevent the associated outbreaks of mosquito-borne infectious diseases [17].

## 2. Materials and Methods

### 2.1. Mosquito Collection and Occurrence

Within the framework of arboviruses surveillance programs, the REVIVE is based on a strategy of collection consisting in monthly to biweekly or even weekly adult and immature mosquito sampling [15]. Adult mosquitoes were caught using Centers for Disease Control miniature light traps (CDC-LT, J. W. Hock Co., Gainesville, FL, USA) baited with CO_2_ set for a minimum of 12 h periods and BG-sentinel traps (Biogents, Regensburg, Germany). Larvae were commonly collected using a dipper in the same localities and also by occasional collections. According to International Health Regulations, ports and international airports have been surveyed by BG-sentinel traps and oviposition traps (ovitraps). These traps consist of an oviposition support (usually germination paper in a wooden stick or a piece of polystyrene) and a small black plastic bucket that is filled with water (0.5–1.5 L) to two-thirds, so that the eggs can be flooded. The mosquitoes were identified using the identification keys of Ribeiro & Ramos [14] and Schaffner et al. [18].

Since 12 June 2017, a tyre company, located in the metropolitan area of Porto (NUTS 3), municipality of Penafiel (LAU 1), parish of Guilhufe and Urrô (LAU 2), was under REVIVE surveillance with two ovitraps and tyre inspections. This parish has a total area of 6.64 km^2^ and 4005 inhabitants (data obtained from Census 2011, Statistics Portugal).

After *Ae. albopictus* detection, larvae prospection was performed in 60 stacked tyres from the company’s tyre discharge area, and adult mosquitoes were collected with an aspirator. In order to define dispersal and abundance, 65 sites on the grounds of the tyre company and in a perimeter of 200 m of the surrounding area were considered to set traps and surveyed for breeding sites. The surveillance methods included the setup of seven BG-sentinel traps and 48 ovitraps, and the inspection of eight gutters and two water tanks. Occasional inspection of other available breeding sites, namely, artificial containers of household waste and flower pots, occurred in the course of the field work. Artificial containers, such as household waste containers, were identified as potential larval sites and were immediately eliminated. Gutters were periodically cleaned since October. Surveillance frequency was weekly from 22 September to 30 November and every two weeks from 1 December. All mosquitoes collected during this period were sent to the laboratory for species confirmation, molecular studies, and pathogen screening.

### 2.2. Molecular Typing

For nucleic acid extraction, mosquitoes were grinded with a mortar and pestle in liquid nitrogen. A volume of 1000 μL of minimal essential medium supplied with 10% FBS, streptomycin (0.1 mg/mL), and amphotericin B (1 mg/mL) was added, and an aliquot of 500 μL was preserved at −80 °C. The remaining volume was further grinded in 300 μL of Lysis Buffer NUCLISENS^®^ easyMAG (BIOMÉRIEUX, Marcy-l'Étoile, France), added to the homogenizer cartridge (Invitrogen, Carlsbad, CA, USA), and centrifuged at 12,000× *g* for 2 min to remove the cellular debris and reduce lysate viscosity. Total nucleic acid extraction was performed using the prepared lysate suspensions in the automated platform NUCLISENS^®^ easyMAG (BIOMÉRIEUX). Molecular typing, using cytochrome oxidase I gene (COI, DNA barcoding region) [19], was immediately performed after morphological identification. In brief, 5 μL of DNA and 10 pmol of each primer were added to FastStart PCR master (Roche, Basel, Switzerland). Polymerase Chain Reaction (PCR) conditions were as follows: denaturation at 95 °C for 3 min, 40 cycles of 94 °C for 20 s, 50 °C for 20 s, and 72 °C for 30 s, and a final extension at 72 °C for 5 min. The obtained amplicons were purified using JETquick PCR Product Purification Spin kit (GENOMED GmbH, Löhne, Germany) and sequenced using ABI Prism 3130 Genetic Analyzer (Applied Biosystems, Foster City, CA, USA). Consensus sequences were edited in BIOEDIT (version 7.0.5.3, Carlsbad, CA, USA) [20], and similarity searches (accession number MF990905) were performed within the GenBank dataset (National Center for Biotechnology Information, NCBI, http://www.ncbi.nlm.nih.gov) using the basic local alignment search tool (BLAST, Rockville Pike, Bethesda, MD, USA) BLASTN algorithm [21].

*Ae. albopictus* sequences obtained from previous studies [22] and accessible from the GenBank database were used as a dataset for a preliminary phylogeographic analysis. The sequences were aligned with ClustalW (Hinxton, Cambridgeshire, UK), and the best-fit model for nucleotide substitution was identified using Mega version 7.0.26 software [23]. Maximum likelihood analysis was performed with the Tamura 3-parameter model, and non-uniformity of evolutionary rates among sites was modelled by using a discrete Gamma distribution among sites (T92+G; the determined best-fit model within Mega 7, Philadelphia, PA, USA). The robustness of the nodes was tested by 1000 bootstrap replications.

### 2.3. Arboviruses Screening

Considering their importance as vectors for several important arboviruses, adult mosquitoes were screened by real-time PCR for chikungunya, dengue, and Zika viruses using the CDC Trioplex Real-time RT-PCR assay (CDC, Atlanta, GA, USA), and by Pan-flavi NS5 conventional RT-PCR to include other potential flaviviruses [24,25].

## 3. Results

**Figure 1:**
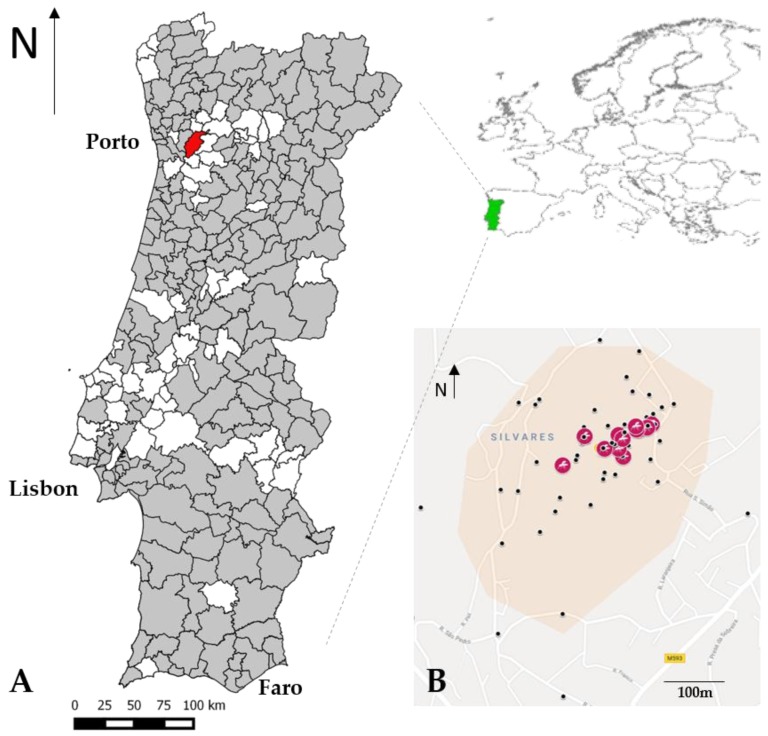
(**A**) In red, the positive location for *Ae. albopictus* in Portugal, municipality of Penafiel (LAU 1), parish of Guilhufe and Urrô (LAU 2) (41°11′08.1′′ N 8°19′45.7′′ W). In grey, the municipalities (LAU 1) surveyed under REVIVE in 2017; (**B**) Distribution map of traps and breeding sites (black points) in the premises of the tyre company and in the 200 m perimeter buffer around the company (rose area). The pink circles indicate the positive sites for the presence of *Ae. albopictus*.

On 4 September 2017, a sample of immature mosquitoes, consisting of five larvae and a pupa, was collected in an ovitrap on the premises of a tyre company in a rural area in the northwest of Portugal, municipality of Penafiel (LAU 1), parish of Guilhufe and Urrô (LAU 2) (Figure 1).

**Figure 2:**
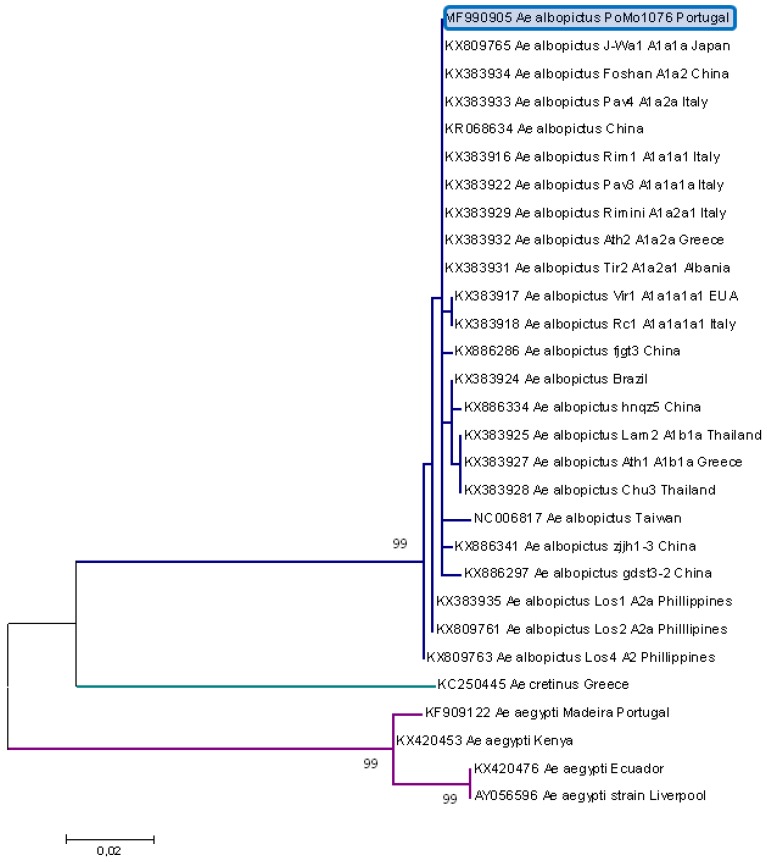
Phylogenetic analysis of *Ae. albopictus* sequences using partial COI sequence region. The maximum likelihood phylogenetic tree was inferred on the basis of 29 partial cytochrome oxidase I (COI) nucleotide sequences (660 bp) by using Molecular Evolutionary Genetics Analysis (MEGA) version 7 software. Distance matrices were calculated using the T92+G model. Bootstrap values obtained from 1000 replicate trees are shown for key nodes (more than 70%). The scale is shown at the bottom as substitutions per site. GenBank accession number and origin are indicated. The *Ae. albopictus* sequence related to this work is highlighted in blue.

The specimens were morphologically identified as *Ae. albopictus* on 8 September 2017. Molecular typing of the specimens (COI, DNA barcoding region) confirmed them as *Ae. albopictus* species (100% identity). The COI partial sequence of 660 bp (accession number MF990905) presented, by BLASTN, a higher similarity to the sequences associated with the Asian haplogroups A1a1 and A1a2, which are found abundantly in the northern hemisphere, namely, in European countries (Figure 2) [22].

INSA laboratory reported the occurrence of *Ae. albopictus* to the DGS on 15 September 2017. Following the inspection, immature mosquitoes were not found in any of the 60 stacked tyres, with water sampled in the tyre company discharge area. However, adult *Ae. albopictus* mosquitoes (four females and a male) were identified from aspiration, indicating a possible establishment of the species in the area. The samples were preserved as voucher specimens.

In the period from 12 June to 20 December, 103 *Ae. albopictus*, including 52 immatures and 51 adults, were collected. Two other mosquito species were collected and identified in the course of the survey, namely, *Culex pipiens* (179 larvae and 5 adults) and *Culiseta longiareolata* (212 larvae). *Ae. albopictus* mosquitoes were only found in the premises of the tyre company. Immatures were mostly collected from gutters, ovitraps, and from one tyre. Adults were mostly collected by aspiration when resting in gutters and ovitraps and foraging for feeding during field activities, but also in BG sentinel traps (Table 1).

**Figure 3:**
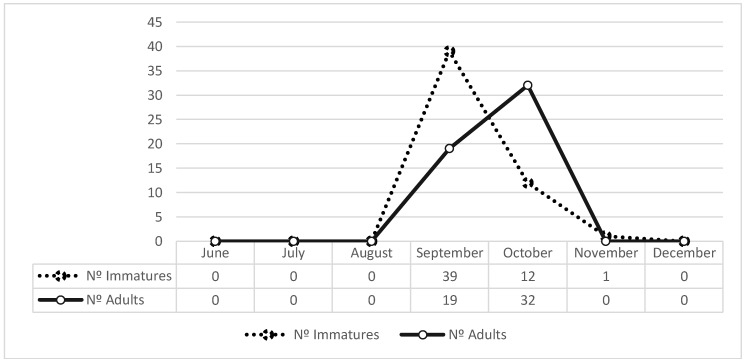
Number of *Ae. albopictus* specimens identified during REVIVE activities at Urrô site from 12 June to 20 December 2017.

Most of the *Ae. albopictus* immatures were collected in September (*N* = 39; 75%), followed by a decrease in October (*N* = 12; 23%). Adults were mostly collected in October (*N* = 32; 63%). The last *Ae. albopictus* sample, a larva specimen, was collected in a tyre on 3 November (Figure 3).

Forty-two adult specimens in 17 pools (containing one to eight females) were screened for the presence of flaviviruses and chikungunya virus RNA. All pools were negative for dengue, Zika, and chikungunya viruses. Although no pathogenic virus RNA was detected, an Insect-Specific flavivirus (ISF) was identified in one pool (one female collected on 4 October) by Pan-flavi NS5 conventional RT-PCR. Sequence similarity analyses ascertained this ISF as more closely related to an *Aedes* flavivirus detected in Italy in 2012 (GenBank accession KF801612).

## 4. Discussion

*Ae. albopictus* was recorded in Portugal in September 2017, where a surveillance programme at the national level has been implemented since 2008. So far, the species was only found at the premises of a tyre company in the parish of Guilhufe and Urrô and at a relatively low number, indicating a population of low abundance, locally introduced, and possibly established, since *Ae. albopictus* was observed breeding also in non-tyre sites, namely, gutters and other artificial containers. With the present data, it is not possible to determine the precise date of introduction, but our data hint at a recent introduction, obviously by tyre importation routes, given the high volume of imported tyres from many international suppliers throughout the year, including Australia, Japan, USA, France, Netherlands, Spain, and England. The risk of dispersal by passive transportation and establishment in neighboring locations is high, considering the particular environmental conditions (namely, the high plant cover of the region and the abundance of water resources and larval habitat). Looking at our survey results, *Ae. albopictus* activity ended early November with no more adult or immature specimens recorded since 3 November. However, the risk of population resettlement in the next spring by overwintering eggs must be considered, and surveillance activities maintained. In addition, the risk of new introductions by tyre importation should always be taken into account.

Tyre companies represent high risk locations for the introduction of invasive mosquito species as a consequence of their commercial activity and are being considered by the REVIVE as points of entry, together with airports, ports, and borders. Procedures for timely detection and efficient surveillance of invasive mosquito species are required by the local authorities in order to avoid the establishment and dispersal of these species and the risk of mosquito-borne disease outbreaks. These procedures should be supplemented with integrated mosquito control measures based on relevant policies in the health sector, such as public health pesticide management and inter-sectorial collaboration, allowing the training of health technicians and physicians in arboviruses surveillance programs and integrated mosquito management.

Although the DNA sequence used in this study was not adequate to determine the origin of the mosquito, the presented phylogenetic analysis indicated a high similarity to sequences associated with the Asian haplogroups A1a1 and A1a2, present in abundance in the northern hemisphere, namely, in European countries, especially Italy, Greece, and Albania (Figure 2) [22]. Moreover, the detection in a mosquito of an *Ae. albopictus* ISF sequence similar to ISF sequences detected in Italy was in agreement with the COI sequences preliminary analysis. ISFs sequences are not available from all European countries where *Ae. albopictus* has been established, which might have induced some bias in this preliminary analysis, but the mutation rate of the flavivirus RNA genome is certainly faster than in mosquitoes’ COI mitogenome, so the detection of an ISF sequence similar to one detected in Italy may induce a suspicion of this European region as the possible origin for Asian tiger mosquito introduction in Portugal. Further studies to assess the origin of the *Ae. albopictus* population in Portugal through mitogenome haplogroup diversity are ongoing.

## 5. Conclusions

The recording of *Ae. albopictus* in the north of Portugal is an important finding outlining a new distribution area of this invasive species in Europe. Surveillance was immediately increased to determine the species expansion and abundance and will be continuously maintained throughout. The preliminary results obtained during September–December showed a relatively low-abundance population present within the perimeter of a tyre company. No pathogenic viruses were detected in the mosquito population. The risk of dispersal and establishment by passive transportation is high, considering the local environmental conditions. To avoid the spread and the establishment of *Ae. albopictus* in other regions, effective surveillance and control measures directed toward eradication should be maintained, considering the high risk of new introductions by tyre importation and/or by mosquito population resettlement in the spring of 2018 by overwintering eggs.

The establishment of this mosquito species in Portugal raises concern for autochthonous transmission of arboviruses, namely, dengue, Zika, or chikungunya viruses, such as the recent outbreak of the latter in Italy, due to the increase in travelling to and from endemic areas [26].

## Figures and Tables

**Table 1:** Number of set traps, screened larval habitats, adult mosquitoes collected by aspiration in the surveillance plan after detection of *Ae. albopictus* (number of positive traps/breeding sites for *Ae. albopictus* in brackets), and specimens collected from 12 June to 20 December 2017.

Trap/Breeding Site (T/BS)	No. of T/BS (Positive T/BS)	No. of Immatures	No. of Adults	Total
BG sentinel	7 (2)		11	11
Flower pots	1 (0)			
Gutter	8 (4)	42	24	66
Ovitrap	48 (6)	9	11	20
Tyre	1 (1)	1		1
Water tank (>1000 L)	2 (0)			
Aspiration			5	5
Total		52	51	103

BG: Biogents sentinel trap.

## Supplementary Materials

The following are available online at http://www.mdpi.com/1660-4601/15/4/820/s1, Table S1: Trap/breeding sites GPS coordinates and positive sites for *Aedes albopictus* mosquitos.
