# Supplementary material for: Detection of the Invasive Mosquito Species Aedes (Stegomyia) albopictus (Diptera: Culicidae) in Portugal
Source: Int J Environ Res Public Health. 2018 Apr 21;15(4):820. doi: 10.3390/ijerph15040820 (PMC5923862; doi:10.3390/ijerph15040820)
Supplement: Supplementary file 1 [file ijerph-15-00820-s001.pdf]

Table S1: Trap/breeding sites GPS coordinates and positive sites for *Aedes albopictus* mosquitos.

Number	Trap/Breeding Site	Lat.	Long.	*Aedes albopictus* Presence
1	BG-sentinel	41.186416°	-8.327547°	Positive
2	Ovitrap	41.186459°	-8.327544°	Negative
3	Ovitrap	41.186423°	-8.327443°	Negative
4	Ovitrap	41.186424°	-8.327637°	Negative
5	Ovitrap	41.186294°	-8.327605°	Negative
6	Ovitrap	41.186552°	-8.327650°	Negative
7	Ovitrap	41.186600°	-8.327725°	Negative
8	Ovitrap	41.186276°	-8.327988°	Positive
9	Ovitrap	41.185875°	-8.328336°	Negative
10	Ovitrap	41.186123°	-8.328778°	Negative
11	BG-sentinel	41.186144°	-8.328674°	Positive
12	Ovitrap	41.186383°	-8.329040°	Negative
13	Ovitrap	41.186356°	-8.329872°	Negative
14	Ovitrap	41.186792°	-8.329498°	Negative
15	Ovitrap	41.186425°	-8.328485°	Negative
16	Ovitrap	41.185582°	-8.328519°	Positive
17	BG-sentinel	41.185603°	-8.328540°	Negative
18	Ovitrap	41.185791°	-8.328681°	Positive
19	Ovitrap	41.185169°	-8.329153°	Negative
20	Ovitrap	41.185124°	-8.328799°	Negative
21	Ovitrap	41.185781°	-8.329159°	Positive
22	BG-sentinel	41.185800°	-8.329204°	Negative
23	BG-sentinel	41.185986°	-8.329737°	Negative
24	Ovitrap	41.185940°	-8.329936°	Negative
25	Ovitrap	41.186109°	-8.329845°	Positive
26	BG-sentinel	41.186096°	-8.329870°	Negative
27	BG-sentinel	41.185490°	-8.330146°	Negative
28	Ovitrap	41.185353°	-8.330588°	Positive
29	Ovitrap	41.185608°	-8.330093°	Negative
30	Ovitrap	41.184529°	-8.330670°	Negative
31	Ovitrap	41.184170°	-8.330835°	Negative
32	Ovitrap	41.183637°	-8.331352°	Negative
33	Ovitrap	41.184326°	-8.329629°	Negative
34	Ovitrap	41.185002°	-8.329215°	Negative
35	Ovitrap	41.186000°	-8.327267°	Negative
36	Ovitrap	41.186695°	-8.327505°	Negative
37	Ovitrap	41.186863°	-8.327213°	Negative
38	Ovitrap	41.186944°	-8.326789°	Negative
39	Ovitrap	41.187181°	-8.327640°	Negative
40	Ovitrap	41.187272°	-8.328202°	Negative
41	Ovitrap	41.187647°	-8.328416°	Negative
42	Ovitrap	41.186936°	-8.331514°	Negative
43	Ovitrap	41.185443°	-8.331477°	Negative
44	Ovitrap	41.184686°	-8.332118°	Negative
45	Ovitrap	41.184735°	-8.332702°	Negative
46	Ovitrap	41.18335º	-8.33265°	Negative
47	Ovitrap	41.17938°	-8.33057°	Negative
48	Ovitrap	41.184110°	-8.324280°	Negative

49	Ovitrap	41.18832º	-8.32799°	Negative
50	Ovitrap	41.1886º	-8.32932°	Negative
51	Ovitrap	41.18698º	-8.33208°	Negative
52	Ovitrap	41.18151º	-8.3306°	Negative
53	Ovitrap	41.18426º	-8.32543°	Negative
54	Ovitrap	41.18493º	-8.32735°	Negative
55	Ovitrap	41.18551º	-8.3276°	Negative
S1	Gutter	41.186342°	-8.327701°	Positive
S2	Gutter	41.186388°	-8.327679°	Negative
S3	Gutter	41.186291°	-8.328062°	Positive
S4	Gutter	41.186362°	-8.328098°	Positive
S5	Gutter	41.186051°	-8.328552°	Positive
S6	Gutter	41.186211°	-8.328484°	Negative
S7	Gutter	41.185901°	-8.328808°	Negative
S8	Gutter	41.185934°	-8.328908°	Negative
T1	Water tank (>1000L)	41.187067°	-8.331394°	Negative
T2	Water tank (>1000L)	41.181005°	-8.332791°	Negative
